# Comparing the Effectiveness of a Wearable Activity Tracker in Addition to Counseling and Counseling Only to Reinforce Leisure-Time Physical Activity among Breast Cancer Patients: A Randomized Controlled Trial

**DOI:** 10.3390/cancers13112692

**Published:** 2021-05-30

**Authors:** Sunga Kong, Jae Kyung Lee, Danbee Kang, Nayeon Kim, Young Mog Shim, Won Park, Dooho Choi, Juhee Cho

**Affiliations:** 1 Department of Clinical Research Design and Evaluation, SAIHST, Sungkyunkwan University, Seoul 06351, Korea; sung00kong@skku.edu (S.K.); dbee.kang@gmail.com (D.K.); 2 Patient-Centered Outcomes Research Institute, Samsung Medical Center, School of Medicine, Sungkyunkwan University, Seoul 06351, Korea; roemgirls1230@gmail.com; 3 Center for Clinical Epidemiology, Samsung Medical Center, Sungkyunkwan University School of Medicine, Seoul 06351, Korea; 4 Cancer Education Center, Samsung Comprehensive Cancer Center, Samsung Medical Center, Sungkyunkwan University School of Medicine, Seoul 06351, Korea; ezhyang.kim@samsung.com; 5 Department of Thoracic and Cardiovascular Surgery, Samsung Medical Center, Sungkyunkwan University School of Medicine, Seoul 06351, Korea; youngmog.shim@samsung.com; 6 Department of Radiation Oncology, Samsung Medical Center, Sungkyunkwan University School of Medicine, Seoul 6351, Korea; wonro.park@samsung.com; 7 Department of Epidemiology and Health, Behavior and Society, Johns Hopkins Bloomberg School of Public Health, Baltimore, MD 21205, USA

**Keywords:** wearable activity tracker, physical activity, breast cancer, radiation therapy, reinforcing factor, step counters

## Abstract

**Simple Summary:**

Many interventions have been introduced to promote PA among patients with breast cancer. While patients have become active and their PA levels have improved during interventions, they discontinued their PA and became inactive once the study ended. Recently, interventions that use new technologies, such as wearable activity trackers (WATs) or smartphones, have become popular and replaced traditional face-to-face interventions emphasizing its efficiency. However, the use of wearable devices would not be sufficient for intervening in PA among patients with breast cancer, as each patient has different physical conditions, baseline PA levels, and clinical characteristics requiring individual counseling. Therefore, we conducted a randomized controlled trial to evaluate whether WAT in addition to counseling would reinforce leisure-time PA (LTPA) among patients with breast cancer under radiation therapy (RT) in comparison with counseling only. We found that the counseling with WAT application group had increased LTPAs immediately after the intervention and they were more likely to maintain long-term LTPAs (6 months after the intervention). We also found that patients who did not perform regular PAs before cancer diagnosis had significantly increased step counts (steps/day) compared with those who did perform regular PAs before cancer diagnosis.

**Abstract:**

This randomized controlled trial aimed to compare the effectiveness of a wearable activity tracker (WAT) in addition to counseling (WAT+counseling) and counseling only for reinforcing leisure-time physical activity (LTPA) among breast cancer patients during radiotherapy (RT). A total of 152 breast cancer patients who were planning to undergo radiation therapy (RT) after surgery participated in the study. The WAT+counseling group (*n* = 76) underwent physical activity (PA) self-monitoring using a WAT and participated in counseling. The counseling-only group (*n* = 76) received telephone counseling once a week during RT and did not receive WAT. The WAT+counseling group had increased relative change in self-reported LTPA (102.8) compared with the counseling-only group (57.8) immediately after RT compared to baseline. Although the relative changes of self-reported LTPA of the WAT+counseling group were higher at three and six months after the end of RT compared to in the counseling-only group, the results were not significant. The mean average daily step count of the WAT+counseling group was 9351.7, which increased to 11,592.2 during RT and 12,240.1 after RT. In the subgroup analysis, patients who did not perform regular PA before cancer diagnosis had significantly increased step counts. This study shows the feasibility of WAT with counseling to reinforce PA among breast cancer patients.

## 1. Introduction

The overall 5-year survival rate for breast cancer is relatively high compared to other cancers (90.8% from 2006 to 2012) [1]. Physical activity (PA) has been linked to increased energy expenditure and reduced incidence of obesity, which affects breast cancer outcomes, including mortality [2]. Specifically, regular participation in moderate-to-vigorous physical activity (MVPA) is associated with diminished treatment side effects, increases in muscle strength and cardiorespiratory fitness, reduced fatigue, enhanced quality of life, and prolonged survival for breast cancer survivors [3,4,5].

Despite these benefits, many breast cancer patients do not meet the American Cancer Society physical activity recommendation of at least 150 min of MVPA per week [6,7]. During primary treatment, only 10% of cancer patients are active, with 20–30% being active post-treatment [8,9]. Most cancer patients experience a significant decrease in physical activity during cancer treatment [10]. Cancer patients often minimize daily activities including job, housework, transportation, or other daily essential activities because they prefer to focus on treatment [11]. To increase MVPA during treatment, cancer patients were advised to participate in more leisure-time physical activities (LTPA) [12], which are defined as PAs that are not essential for daily living and are performed at the discretion of the person. Examples include sports, exercise, and recreational walking, all of which are usually MVPA [13].

Many interventions have been introduced to promote PA among breast cancer patients [14,15]. However, a majority of these interventions include face-to-face supervised programs and regular visits, which require significant resources [16]. While patients became active and increased PA levels during interventions, they discontinued their PA and resumed inactivity once the study concluded [17].

Recently, interventions that use new technologies, such as the wearable activity tracker (WAT) or the smartphone [18,19], have become popular in the general population or in patients with other chronic diseases, such as diabetes mellitus [20]. New devices and technologies help individuals stay active by providing continuous support, such as bio-feedback monitoring and reminder functions [18,21]. According to the transtheoretical model (TTM), maintenance of a healthy behavior is associated with a reinforcing factor, including continuous support [22], which might help patients achieve long-term behavioral change [23], and this is one of the benefits of the WATs when compared to types of interventions such as individual support interventions. However, the use of WATs alone would be insufficient for PA intervention [24,25]. Each patient has different physical conditions, baseline PA levels, and sociodemographic factors, all clinical characteristics requiring individual counseling [26]. Conversely, individual counseling to reinforce daily PA requires significant time and resources [27]. Therefore, researchers designed a RCT using a WAT plus telephone counseling intervention to increase MVPA and reduce sedentary behavior in breast cancer survivors [28]. They found that the intervention helped breast cancer survivors to achieve significant changes in MVPA and sedentary behavior. However, in this study, the control group received no intervention, and it was difficult to determine whether the effectiveness of the intervention was due to WAT or the telephone counseling. In addition, most previous studies were conducted with a small number of participants (<50) [25] and limited to survivors who completed treatment [28]. Thus, we conducted an RCT to evaluate whether a WAT in addition to face-to-face counseling (WAT+counseling), as opposed to counseling only, would reinforce and increase recommended levels of LTPA in breast cancer patients.

## 2. Materials and Methods

### 2.1. Trial Design and Participants

This study was a single-center randomized controlled trial conducted to compare the effectiveness of WAT+counseling and counseling-only for reinforcing LTPA among breast cancer patients. The study was approved by the Clinical Research Information Service (https://cris.nih.go.kr/cris): approval number KCT0001474, approved on 18 March 2015. The study was conducted from May 2015 to December 2017 at the Samsung Comprehensive Cancer Center in Seoul, South Korea. We decided to focus on patients receiving RT considering the relevance of PA and feasibility of the study. While PA is recommended during all cancer treatments, moderate-to-vigorous activity is often not feasible for breast cancer patients coping with surgery or chemotherapy [29]. In addition, fatigue is the most prevalent and severe problem breast cancer patients reported during RT, which can be improved by PA [4]. In terms of feasibility of the study, when we conducted the trial, WATs were new to the market and most middle-age or older adults were unfamiliar with the technology and, if left unassisted, may fail to use the device [30]. As RT patients require daily hospital visits, we would be available to provide technical assistance for the proper use of the WAT.

The eligibility criteria were as follows: age of 19–65 years, stage I-III breast cancer, and plan to undergo RT after surgery, Eastern Cooperative Oncology Group Performance Status score of <2, and ownership of a smartphone. We excluded patients who were unable to walk or needed assistive walking devices; who had a history of treatment for central nervous system disorders and severe neuropsychiatric disorders (dementia, severe depression, and mental disease); who had metastatic or recurrent disease; and who were unable to communicate in Korean. Participants were recruited by medical record review, physician referral, and posters when they were prescribed to undergo RT (1 to 3 weeks before RT).

Patients who agreed to participate (*N* = 167) were asked to wear a Fitbit Flex (Fitbit Inc., San Francisco, CA, USA) for 1 week to assess baseline PA level, then return the device using a pre-paid envelope [31]. The Fitbit Flex used for baseline assessment was a simple band type, which does not have a screen to show user feedback to minimize impact on baseline PA results. While the researchers explained the basic purpose of the device to participants, they avoided detailed explanations such as how to use it or how to access data to avoid potential bias. In addition, we did not install the companion app on participant smartphones, and turned off all WAT user feedback functions.

The study was approved by the Institutional Review Board of Samsung Medical Center. All study participants provided written informed consent. An independent monitoring committee reviewed the accumulating safety data throughout the trial. The participants were advised to report any adverse effects during the intervention; however, none were reported.

### 2.2. Intervention

The intervention included the use of the WAT in addition to weekly face-to-face counseling by a doctoral-level exercise physiologist, taking place during the 5 week radiotherapy treatment period. The counseling-only group received weekly telephone counseling from the same physiologist responsible for the intervention group. Telephone counseling was used instead of face-to-face counseling to minimize interaction between the control and intervention groups at clinics [32,33]. At the start date of RT, both groups received counseling from the physiologist. The physiologist reviewed patient baseline PA data (steps taken) obtained using the Fitbit Flex, regular PA before diagnosis, self-motivation, self-efficacy, health status including comorbidity, and cancer treatment history. At the first counseling session, the physiologist asked questions to determine the stage of change [34], and provided counseling following the transtheoretical model (TTM), encouraging patients to begin or maintain PA [35]. The physiologist had 15 years of experience providing TTM-based counseling. Two education booklets (“Guidebook for healthy life after breast cancer treatment” and “Exercise after breast cancer”) were also provided after counseling to both the intervention and control groups. The Guidebook includes general information about stress management, exercise and diet after treatment, sleep management, menopausal symptoms management, sexual life after treatment, numbness management, health examination, and spiritual well-being after treatment.

We used the Fitbit Charge (Fitbit Inc., San Francisco, CA, USA) for the WAT+counseling group, which has a monitor displaying steps taken, energy consumed, and distance traveled [36]. The intervention was applied throughout the 5 week RT treatment period. Once patients were assigned to the WAT+counseling group, they were asked to wear the WAT at all times for use in self-monitoring and promoting their own PA. Additionally, patients were encouraged to use the companion smartphone app to maximize the effectiveness of the WAT. Research personnel taught patients how to use the WAT and smartphone app, and provided a user manual for the system. The researchers encouraged the WAT+counseling group participants to view their data at least twice a day, e.g., in the morning and late afternoon or evening. The activity tracker was set to vibrate or flash when the patients reached their daily goal.

The counseling sessions consisted of an evaluation of participants’ activity level, review of the major achievement or challenges of doing LTPA, and goal setting for the next week (increased, maintained, or reduced) considering the patient’s overall health status, treatment-related symptoms, and feedback about LTPA. At the first counseling session, the physiologist set up an initial goal based on patient baseline PA. Target goal setting was designed with the following principles: 1000-step increments are congruent with 10 min walking bouts at 100 steps/min or minimally moderate-intensity PA (LTPA), and three 10 min bouts (3 × 1000 steps = 3000 steps) are congruent with a daily 30 min LTPA for cancer patients [37,38]. The increase or decrease of the weekly goal was set within 3000 steps (or 30 min) of the maximum, according to the FITT-VP of the ACSM exercise guideline [38]. However, all goal decisions were made with patient agreement. If patients did less than 150 min LTPA per week at baseline, the physiologist asked patients to take an additional 3000 steps on more than 5 days a week [37,38]. If a patient participated in LTPA for more than 150 min per week, the physiologist requested an additional 1000–3000 brisk steps the following week. If a patient participated in more than 300 min LTPA per week at baseline [39], the physiologist asked them to maintain their current PA level. Starting with the second counseling session, the previous week’s record was reviewed with patients and the physiologist modified the goal (increased, maintained, or reduced) considering the patient’s overall health status, treatment-related symptoms, and feedback about LTPA. Once a goal was achieved, the physiologist asked patients to take an additional 1000–3000 brisk steps the following week (up to 12,000 steps per day) [40,41]. For patients who did not achieve the goal of the preceding week, the physiologist modified the goal (mean of last week’s goal and the patient’s actual steps completed) and asked the patient whether they agreed upon the new goal. If a patient expressed concern about achieving the suggested goal due to physical condition, the physiologist asked the patients to try to achieve a minimum requirement, which was 3000 brisk steps, five days a week. For the counseling-only group, the same algorithm was used but with time (minutes) instead of steps (1000 steps in 10 min) [6,38,42]. After the intervention period, patients returned the WAT, and counseling was discontinued.

### 2.3. Outcomes

The primary outcome was the relative change in self-reported LTPA levels between baseline measurements and measurements taken immediately following RT. We used the Global Physical Activity Questionnaire (GPAQ) to measure LTPA levels [43,44]. Among the GPAQ items, we used the following each 3 items to calculate the sum of vigorous- and moderate-intensity LTPA: (a) Do you do any vigorous-intensity (moderate-intensity) sports, fitness, or leisure (recreational) activities that cause large (small for moderate-intensity) increases in breathing or heart rate?; (b) In a typical week, on how many days do you do vigorous-intensity (moderate-intensity) sports, fitness, or leisure (recreational) activities?; (c) How much time do you spend doing vigorous-intensity (moderate-intensity) sports, fitness, or leisure activities on a typical day?. We measured the self-reported LTPA before RT, immediately after RT, and 3 and 6 months after completion of RT.

For the WAT+counseling group, the average daily step counts during RT, which was measured using the WAT, was also evaluated. The data obtained from the WAT were downloaded by log-in to each patient’s account on the Fitbit website (Available from URL: https://www.fitbit.com/settings/data/export). The equivalence of the WAT we used for baseline (Fitbit Flex) and intervention (Fitbit Charge) has been well validated and it could be interchangeable [45,46]. In addition, WAT compliance was assessed based on data recorded by the WAT (number of steps per day). If the data indicated 0 steps per day, we determined that the patient did not wear the WAT that day. Among the WAT+counseling group, the Fitbit compliance mean was 96.1 ± 7.0% (range 66.7~100).

### 2.4. Other Variables

All participants completed the questionnaire before RT by themselves. The self-motivation for PA was measured using the Korean version of the self-motivation assessment scale [47]. It contains seven items in a five-point Likert scale to determine individuals’ initial motivation and proneness to non-compliance. A summed total score of ≤24 points indicates a higher likelihood of quitting exercise. The Korean version of the general self-efficacy scale was used to assess self-efficacy [48,49]. A higher total score of the 10 items rated in the four-point Likert scale represents higher self-efficacy.

Sociodemographic information, including age, marital status, educational level, working status, drinking status, smoking status, and menopausal status, was recorded before RT using a questionnaire. PA behavior information was also collected including regular PA before diagnosis (“more than once in a week” for yes) [50] and plan to perform PA during cancer treatment (“willing to perform PA with plan” for yes). Body mass index (BMI) was assessed by bioelectrical impedance analysis (Inbody 770; Inbody Co., Ltd., Seoul, Korea). Clinical information, including comorbidities, disease stage, type of surgery, hormone therapy, targeted therapy, and dose of RT, was obtained from electronic medical records. When they had difficulties in understanding or filling out the questions, a researcher helped them complete the questionnaire.

### 2.5. Sample Size

We expected a medium effect size in this study examining differences between two groups, the required sample size to achieve power of 0.80 with alpha of 0.05 is *n* = 63 per group when using Cohen’s (1988) estimate of Cohen’s *d* = 0.50. Considering 15% loss to follow-up, the sample size was 150 (75 per arm).

### 2.6. Randomization and Blinding

The patients were randomized in a 1:1 ratio into the counseling with the WAT+counseling or counseling-only group after assessing the baseline PA level. Randomization was performed using random permuted blocks, and stratification was conducted according to hormone therapy (yes or no) and type of surgery (total mastectomy or partial mastectomy). The random allocation sequence was generated using the Stata 14.0 (Stata Corp., College Station, TX, USA) by a statistician who was not involved in the recruitment procedure. The block size was 4 and 6 to avoid participant allocation predictability as in single-size block allocations. The sequentially numbered opaque envelopes were concealed from the research personnel who enrolled and assigned the participants to the groups. The concealed envelope with randomization allocation was provided to the researchers 1 day before the patients’ visit, and the researchers opened the envelope once the patients completed all of the baseline assessment.

Both patients and researchers could not be blinded because of the nature of the intervention. However, the assessors and statisticians who developed the random blocks and conducted the statistical analysis were blinded to minimize bias.

### 2.7. Statistical Analysis

All analyses were conducted using the intention-to-treat principle, whereas the study patients were assigned to their randomized group irrespective of compliance with the study intervention. For the analysis of the primary outcome, we calculated the relative change in self-reported LTPA immediately after RT. We calculated differences between before RT and follow-up LTPAs and divided them by the absolute value, which measured before RT to determine the relative change. The relative change was calculated using the formula [Relative change (***x***, ***x***_baseline_) = Actual change/***x***_baseline_ = Δ/***x***_baseline_ = ***x*** − ***x***_baseline_/***x***_baseline_].

Because the relative change cannot not be calculated if patients reported 0 min of self-reported LTPA at baseline, we replaced the denominator 0 with 1 to calculate the relative change. However, when the denominator is 1, it cannot affect the actual change. We compared the relative change of self-reported LTPA in the WAT+counseling and control-only groups using *t*-tests. Additionally, we calculated self-reported LTPA differences between baseline measurements and measurements taken 3 and 6 months after RT. We also used a mixed model to test the homogeneity of the slope changes in the mean and ratio of self-reported LTPA before RT, immediately after RT, and 3 and 6 months after RT to assess how the intervention affected the changes. In the subgroup analysis, changes of self-reported LTPA and average daily step counts were also compared using *t*-test and mixed model. The model used case-wise deletion for missing data. In addition, we performed stratified analyses to evaluate the impact of WAT+counseling on the relative change in self-reported LTPA among prespecified subgroups defined by age (<50 vs. ≥50 years), body mass index (<25 vs. ≥25 kg/m^2^), educational level (<college vs. ≥college), disease stage (stages I and II vs. stage III), type of surgery (lumpectomy vs. mastectomy), hormone therapy (no vs. yes), targeted therapy (no vs. yes), regular PA before diagnosis (no vs. yes), plan to perform PA during cancer treatment (no vs. yes), motivation for PA (<24 vs. ≥24 points), and self-efficacy (<28 vs. ≥28 points). We conducted subgroup analysis as these are well-known factors associated with PA among breast cancer survivors. Up to three statistically significant interaction tests (*p* < 0.05) would be expected on the basis of chance alone. Differences in the baseline demographic and clinical characteristics were compared using the Chi-square tests for categorical variables and *t*-tests for continuous variables. All analyses were conducted using the Stata 14.0. *p*-values of <0.05 were considered statistically significant.

## 3. Results

### 3.1. Characteristics of the Study Population

Among the 240 eligible subjects, 167 (69.6%) subjects consented to participate, and 152 patients completed the baseline assessment. These 152 patients were randomly assigned to the counseling with the WAT+counseling (*n* = 76) and counseling-only group (*n* = 76).

**Figure 1:**
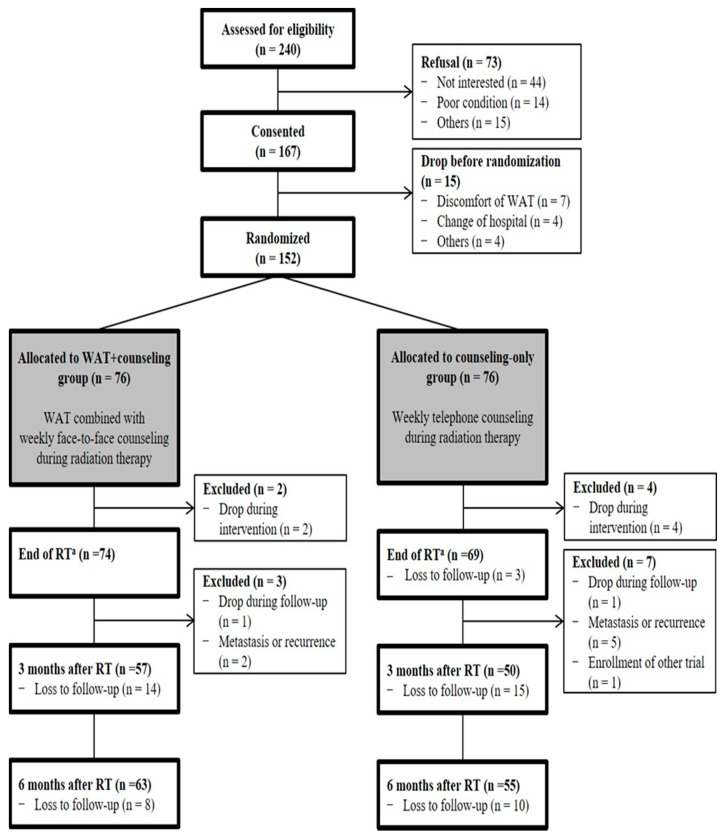
Study flow diagram. The number of participants at each time point reflects those who had complete questionnaire data. Participants who dropped out, had metastasis/recurrence, or enrolled in other trial were permanently excluded from the study, whereas other participants remained in the study and were eligible for measurement at the next time. ^a^ Primary endpoint; RTx, radiation therapy, WAT, wearable activity tracker.

Of the 152 patients, 143 (94.1%; counseling-only group, *n* = 69; WAT+counseling, *n* = 74), 107 (70.4%; counseling-only group, *n* = 50; WAT+counseling, *n* = 57), and 118 (77.6%; counseling-only group, *n* = 55; WAT+counseling, *n* = 63) patients completed the survey at the end of RT and 3 and 6 months after RT, respectively. We excluded nine patients who were loss to follow-up and whose GAPQ were not available. The final analysis for the primary outcome included 143 patients (Figure 1).

The mean age of the study patients was 47.0 years, and 40.8% of the patients had stage III breast cancer. Approximately 35.5% of the patients performed regular PAs before diagnosis, and 75% had a plan to perform PA during treatment. The two groups were similar with respect to regular PA before diagnosis, plan to perform PA during cancer treatment, and motivation and self-efficacy for PA before RT (Table 1).

### 3.2. Relative Change in Self-Reported Leisure-Time Physical Activity

The mean self-reported LTPA (SD) before RT was 105.7 (166.7) and 119.7 (205.4) minutes per week in the counseling-only and WAT+counseling groups, respectively (*p* = 0.64). Self-reported LTPA increased in both groups at follow-up. At the end of RT, 181.7 and 226.4 min/week of self-reported LTPA were conducted by the counseling-only and WAT+counseling groups, respectively. The corresponding values of relative change were 57.8 and 102.8, respectively (*p* = 0.03). The WAT+counseling group was more likely to have increased self-reported LTPA at three and six months compared to baseline than the counseling-only group; however, the findings were not significant (Table 2).

**Figure 2:**
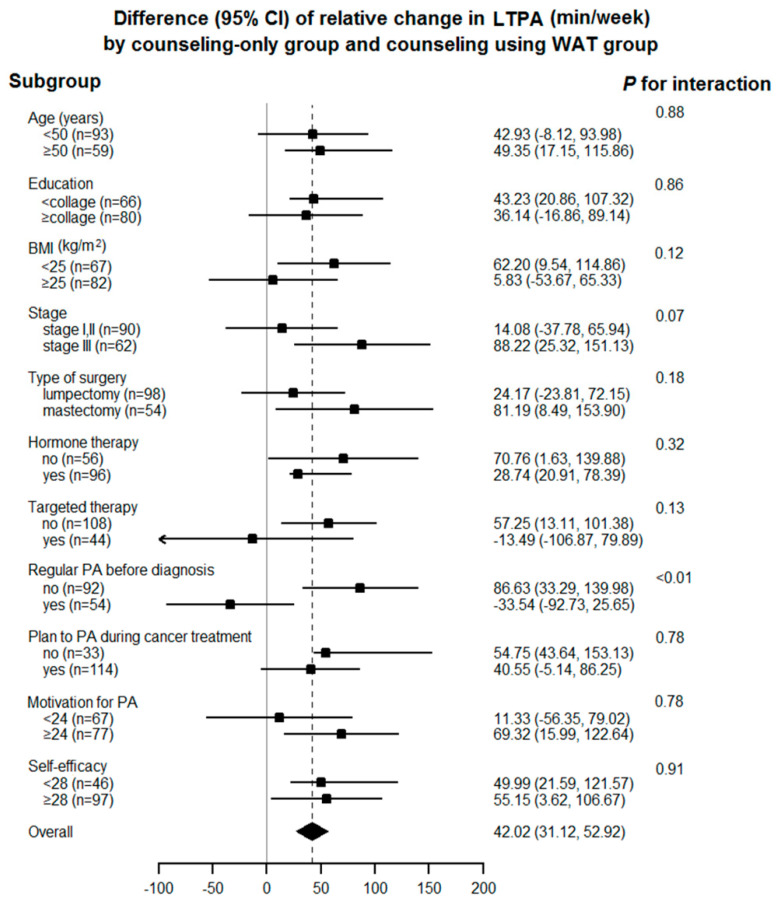
Subgroup analysis of relative change in self-reported leisure time physical activity at end of radiation therapy.

The range of step data for the WAT+counseling group during the intervention was 1279~32,404 steps per day. In the subgroup analysis, we found that the association was stronger in the patients who did not perform regular PA before diagnosis than in the patients who did (*p* for interaction, <0.01; Figure 2).

**Figure 3:**
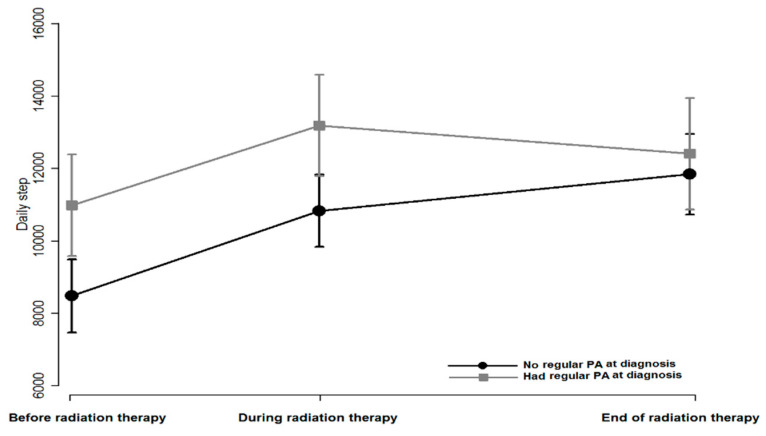
Average daily steps in WAT+counseling group PA, physical activity.

Among the WAT+counseling group, the mean average daily step count was 9351.7, which increased to 11,592.2 during RT and to 12,240.1 after RT (Table 3). While the mean average daily step count (SD) before RT (before randomization) between the patients who did not perform (8336.6, SD = 3326.1) and who performed regular PA (11,012.4, SD = 5025.3) before diagnosis was significantly different (*p* = 0.013), it was similar at the end of the intervention [11,930.9 (502.6) and 12,766.3 (917.0) for the patients who did not perform and who performed PA, respectively] (*p* = 0.39; Figure 3).

## 4. Discussion

In this trial, we found the relative change in self-reported LTPA of the WAT+counseling group immediately after the intervention was larger compared to baseline than those of the counseling-only group. Although the relative changes of self-reported LTPA were higher at 3 and 6 months after the end of RT than the counseling-only group, the results were not significant. In particular, the intervention was found to be more effective in patients who did not perform regular PA before diagnosis. These results demonstrate the possibility of using WATs as a reinforcing factor to encourage PA during RT among breast cancer patients.

While the outcome is limited to the intervention group, despite the relatively short intervention period of the present study, the step count for the WAT+counseling group increased by an average of approximately 2900 steps/day during the intervention period. This is consistent with the findings of previous studies, but with a larger effect. An intervention study (*n* = 40) that evaluated the effectiveness of a WAT plus telephone counseling found that breast cancer survivors who were post-menopause at diagnosis had increased PA compared to the control group, which did not receive additional intervention following during a previous 12 week intervention [28]. Another study (*n* = 51) found that postmenopausal overweight women who used Fitbit averaged an 800-step increase after 16 weeks of intervention, while the control group did not show an increase in step count [51]. The larger impact might be attributed to participant characteristics. Our study participants were younger than those of previous studies, and they might be more motivated to perform PA. Additionally, our study participants were receiving RT while other interventions were conducted with survivors who had completed treatment. A larger study with heterogeneous participants will be necessary in the future.

In our study, patients in the WAT+counseling group had significantly more increased self-reported LTPAs than the counseling-only group. The WAT seems to encourage physical activity and exercise by reminding patients of their PA status, thus boosting the counseling effect. The WAT seems to encourage physical activity and exercise by reminding patients of their PA status, thus boosting the counseling effect. In fact, researchers found that WATs give users a sense of control by providing real-time feedback and serving as a self-monitoring system [52]. Considering the reasonable price and portability of the device [53], WAT+counseling might be a good intervention tool to encourage breast cancer patients to stay active on a long-term basis. However, it is necessary to evaluate the long-term effect of the intervention, as our study was performed over a relatively short period.

In our study, there was an effect of maintaining physical activity until 6 months after completion of the intervention in the WAT+counseling group. The WAT+counseling group might have long-term effects when it comes to maintaining increased levels of LTPA compared to the counseling only group. Since WATs help individuals to understand their PA pattern and increase it by showing the step count during intervention, the WAT+counseling group might learn how to self-monitor their LTPA level and keep them high even after the intervention has ended. On the other hand, the group undergoing counseling alone showed increased LTPA levels at 3 months after RT and then decreased levels at 6 months after RT. According to a systematic review and meta-analysis of long-term PA behavior, on-site supervised, individual-support interventions can successfully increase PA in the short and medium term [54] and have a moderate impact at least 3 months post-intervention completion [55]. In this study, WAT may aid in maintaining motivation to be physically active in individuals after the novelty of initiating an activity program has waned. However, given the low evidence quality, further methodologically rigorous studies are warranted to evaluate the long-term effects.

WAT+counseling might have a greater effect, as the counseling was tailored to the needs of each study participant. Previous studies suggest that constructs from the TTM are associated with greater motivation for PA [56]. Based on the findings of available empirical studies, the reinforcing factors in the PRECEDE–PROCEED model for health promotion are all relevant and important for the adoption and maintenance of PA [57]. The TTM explains behavioral changes for promoting long-term PA [58] in five stages: (a) precontemplation; (b) contemplation; (c) preparation; (d) action; and (e) maintenance. The first two stages identify unwillingness/scarce preparation to change toward healthier behaviors, and the last three stages indicate readiness to change or active involvement in healthy lifestyle [59]. In our study, counseling was included to determine the PA status of each patient and help them understand their PA status; further, the WAT was designed for supporting the patients to change or maintain their PA depending on their PA level before RT. In fact, the patients who did not perform regular PA had an increased LTPA after the intervention and maintained such over time. To maintain long-term PA, reinforcing factors are necessary [60], and the WATs’ supporting and monitoring functions in home-based PA programs could be good reinforcing factors for populations who need continuous support and help.

In our study, the WAT+counseling group maintained a similar level of self-reported LTPA over time and stayed active 3 months and 6 months after the intervention. Generally, PA decreases during treatment (chemical and/or radiation therapy) and increases after treatment [61]. In our study, there was no change in self-reported LTPA for the WAT+counseling group as the PA level in that group was already high at the end of RT. Most intervention studies focus on creating sustained increases in regular PA behaviors [62]. Nevertheless, previous studies have shown the limited effect in maintaining PA after the intervention [63]. While most patients require long-term support and monitoring [64], it is difficult for health professionals to supervise them continuously owing to limited time and resources [65]. WATs could be one of the supporting devices that could yield benefits to both patients and health professionals. However, more studies are necessary to support their use in clinical settings, as patients would prefer traditional delivery modalities (e.g., face-to-face or telephone methods) over technology-based methods (e.g., computer or activity trackers) [24,51]. Conversely, the effect might be different depending on the behavioral target of the intervention.

Among the WAT+counseling group, the patients who did not perform regular PA had significantly increased self-reported LTPAs compared with those who did. Before RT, there was an approximately 2600-step difference between the patients who performed and did not perform regular PA (*p* = 0.013); however, there was no difference in the steps/day between the groups at the end of the intervention. According to the literature, individuals who did not perform PA need more practice and familiarization to be active, as they had low self-efficacy, belief, and self-regulation strategies for PA [66]. WATs might help inactive individuals to understand their PA pattern and practice it by showing the step count and activity status. It is possible that WATs might be more attractive to patients who did not perform regular PA because they remind them to be active [67]. This can be one of the strengths of WATs, as they could help patients regarding continuous self-monitoring and allow users to have a sense of control by providing real-time feedback [68].

This study has several limitations. First, the intervention was conducted in a period shorter than those in previous studies. Nonetheless, we investigated the long-term effects after the intervention. Second, we evaluated PA using the GPAQ with acknowledgment of the potential bias inherent in self-reported PA outcomes. However, previous studies reported the reliability and validity of the GPAQ [43], and researchers have used it for evaluating the effectiveness of interventions to promote MVPA [44]. Third, in this study, the same physiologist provided counseling to both the WAT+counseling and counseling-only group without blind, and it might introduce bias. To minimize the bias, the physiologist performed the counseling using standard protocol, and the final analysis was conducted by a statistician who was not involved in this study. Fourth, we compared phone counseling sessions as control to face-to-face counseling sessions using WAT, and the effects of WAT might be a difference in the effectiveness of counseling delivered by phone and in person. However, we decided to provide telephone counseling instead of face-to-face counseling to minimize interaction between the control and intervention groups at clinics [32,33]. Fifth, there were 9 patients who had missing items on the primary end points and were omitted in the analysis, but the proportion of patient with missing and the baseline self-reported LTPA were similar between intervention and control. Sixth, while WAT could be used for individualized PA intervention regardless of type of cancer or treatment [69], the individual PA counseling should be performed considering cancer- and cancer treatment-related factors. However, the study included only patients who were scheduled to receive RT for breast cancer at a single center, which may limit the generalizability of the findings.

Thus, future studies should evaluate the approaches tested in the present study among samples that are more diverse, such as old patients, patients with advanced stage, and patients with concurrent chemotherapy or radiotherapy. In addition, a comparison of whether the decrease fatigue, reduce body fat, improve cardiovascular function effects is needed to evaluate the usefulness of the WAT.

## 5. Conclusions

This randomized controlled trial suggests that WAT in addition to counseling was effective in improving the PA of breast cancer patients during RT. However, additional studies with larger sample sizes with longer follow-up periods are necessary to confirm the benefits of WATs in terms of reinforcing PA.

## Figures and Tables

**Table 1:** Baseline characteristics.

	Counseling-Only Group (*N* = 76)	WAT+Counseling Group (*N* = 76)	*p*-Value
Age (years)	46.8 ± 7.6	47.3 ± 8.5	0.67
Marital status (married)	63 (82.9)	59 (77.6)	0.42
Educational level (≥college)	44 (57.9)	39 (51.3)	0.42
Employment status before diagnosis (employed) ^a^	46 (60.5)	47 (61.8)	0.87
Drinker for the past 1 year (yes)	38 (50.0)	30 (39.5)	0.19
Smoker for the past 1 year (yes)	6 (7.9)	5 (6.6)	0.75
Body mass index (kg/m^2^)	24.0 ± 3.1	23.8 ± 3.4	0.75
Menopausal status at diagnosis (premenopause)	53 (69.7)	46 (60.5)	0.23
Comorbidity (yes) ^b^	32 (42.1)	25 (32.9)	0.24
Stage			0.61
I	13 (17.1)	15 (19.7)	
II	34 (44.7)	28 (36.8)	
III	29 (38.1)	33 (43.4)	
Type of surgery (Lumpectomy)	50 (65.8)	48 (63.2)	0.73
Reconstruction surgery (yes)	6 (7.9)	6 (7.9)	0.99
Chemotherapy (neoadjuvant)	55 (72.4)	46 (60.5)	0.12
Dose of radiation therapy (cGy)			0.06
5000–6000	43 (56.6)	54 (71.1)	
6000–6500	33 (43.4)	22 (29.0)	
Hormone therapy (yes, %)	49 (64.5)	47 (61.8)	0.74
Regular PA before diagnosis (yes, %)	29 (38.2)	25 (32.9)	0.79
Plan to perform PA during cancer treatment (yes, %)	55 (72.4)	59 (77.6)	0.58
Motivation for physical activity ^c^	22.8 ± 4.3	22.0 ± 4.0	0.29
Self-efficacy ^d^	28.0 ± 3.1	28.0 ± 4.8	0.99

Values are presented as means ± SDs or numbers (%). ^a^ Employed status was defined as working for more than 1 h/week for an income or working for more than 18 h/week as an unpaid family worker. ^b^ Comorbidities included cerebrovascular disease, cardiovascular disease, diabetes mellitus, pulmonary disease, musculoskeletal disease, liver disease, hypertension, renal disease, ocular disease, gynecologic disease, and other diseases. ^c^ Motivation for physical activity was measured using the self-motivation assessment scale. ^d^ Self-efficacy was measured using the general self-efficacy scale. PA, physical activity; WAT, wearable activity tracker.

**Table 2:** Relative changes in self-reported LTPA by group.

	Counseling-Only Group (*N* = 76)	WAT+Counseling Group (*N* = 76)	*p*-Value
Self-reported LTPA (minute/week)			
Before RT	105.7 ± 166.7	119.7 ± 205.4	0.64
Immediately after RT ^a^	181.7 ± 191.0	226.4 ± 238.3	0.22
3 months after RT	202.0 ± 299.1	227.3 ± 241.8	0.63
6 months after RT	194.0 ± 240.9	220.0 ± 254.2	0.57
P for trend	0.04	0.02	
Relative changes in self-reported LTPA ^b^			
Before RT	Reference	Reference	
Immediately after RT ^a^	57.8 (32.1, 83.5)	102.8 (72.3, 133.4)	0.03
3 months after RT	86.6 (7.14, 166.0)	99.0 (50.7, 147,4)	0.78
6 months after RT	56.6 (20.8, 92.4)	94.5 (56.0, 133.2)	0.15

^a^ Primary endpoint; ^b^ Relative changes were calculated as the self-reported LTPA change between each time point and value before RT in the value divided by the absolute value, which was measured before RT. LTPA, leisure-time physical activity; RT, radiation therapy; WAT, wearable activity tracker.

**Table 3:** Average daily step count (steps/day) in the WAT+counseling group during RT.

	**Total**	**Regular PA before Diagnosis**		*p*-Value
		No	Yes	
Before RT (*n* = 68)	9351.7 ± 4273.2	8336.6 ± 3326.1	11,012.4 ± 5025.3	0.01
During RT (*n* = 75)	11,592.2 ± 3289.8	10,858.5 ± 2836.0	13,136.0 ± 3692.7	<0.01
Immediately after RT (*n* = 74)	12,240.1 ± 3757.9	11,930.9 ± 502.6	12,766.3 ± 917.0	0.39
*p* for trend	<0.01	<0.01	0.13	

WAT, wearable activity tracker; PA, physical activity; RT, radiation therapy.

## Data Availability

Not applicable.

## Abbreviations

PA: physical activity; RT: radiation therapy; WAT: wearable activity tracker; TTM: transtheoretical model; LTPA: leisure-time physical activity; GPAQ: Global Physical Activity Questionnaire.
